# Natural bioactive compounds: a potential therapeutic strategy to sensitize bladder cancer to cisplatin treatment?

**DOI:** 10.20517/cdr.2022.02

**Published:** 2022-04-06

**Authors:** Vicenç Ruiz de Porras

**Affiliations:** ^1^Catalan Institute of Oncology, Badalona Applied Research Group in Oncology (B·ARGO), Ctra. Can Ruti-Camí de les Escoles s/n, Badalona 08916, Spain.; ^2^Germans Trias i Pujol Research Institute (IGTP), Ctra. Can Ruti-Camí de les Escoles s/n, Badalona 08916, Spain.

**Keywords:** Bladder cancer, muscle-invasive bladder cancer, cisplatin, chemoresistance, natural products, curcumin, bioavailability

## Abstract

Bladder cancer (BC) is the tenth most common cancer, and its incidence is steadily rising worldwide, with the highest rates in developed countries. Neoadjuvant cisplatin-based chemotherapy followed by radical cystectomy is the standard therapy for patients with muscle-invasive bladder cancer. However, less than 50% of patients initially respond to this treatment and nearly all of them eventually develop resistance, which is an important barrier to long-term survival. Therefore, there is an urgent need to understand the mechanisms of cisplatin resistance in BC and develop ways to counteract them. Several preclinical studies have demonstrated that naturally derived bioactive compounds, such as phytochemicals and flavonoids, can enhance the antitumor activity of cisplatin, with minimal side effects, by targeting different pathways involved in cisplatin sensitivity and resistance. However, their poor bioavailability has been one of the main problems for their successful introduction into clinical practice. At present, however, many new formulations with greatly increased bioavailability are under study in several clinical trials with encouraging results.

Bladder cancer (BC) is a current clinical problem due to its high incidence, prevalence, and mortality^[1,2]^. At diagnosis, 70% of patients present with non-muscle-invasive BC, a highly recurrent tumor treated by transurethral resection followed by BCG instillation. However, a high proportion of patients have recurrence and progress to muscle-invasive BC (MIBC), which is also present in 30% of patients at diagnosis^[3]^. At present, the treatment of MIBC has several limitations. Cisplatin-based neoadjuvant chemotherapy followed by radical cystectomy is the standard therapy, with the highest level of evidence, but it is only effective in 25%-50% of patients and confers a limited survival benefit^[4]^. Moreover, this survival benefit is only observed in patients with pathological response, while the delay in cystectomy in those who do not respond has a negative prognostic impact, highlighting the need for predictive biomarkers to identify those patients likely to benefit from cisplatin-based neoadjuvant treatment^[5]^. In addition, adjuvant chemotherapy has been recommended for patients with high-risk MIBC, although randomized trials have not provided conclusive evidence on the impact of this approach^[6-8]^. Furthermore, nearly 50% of patients will develop metastatic disease after undergoing radical cystectomy^[3]^, and platinum-based chemotherapy is considered the cornerstone of treatment for these patients as well^[9]^. Nevertheless, immunotherapy - especially immune checkpoint inhibitors (ICIs) - are potentially effective in metastatic BC (mBC)^[10,11]^, although only 20%-30% of mBC patients respond and there are no reliable predictive biomarkers of response^[12]^. Importantly, with the approval of ICIs for the treatment of mBC, they are now being studied in the neoadjuvant setting for MIBC with promising results^[13]^. Thus, based on this evidence, it is clear that cisplatin still plays an essential role in the treatment of MIBC. However, less than 50% of patients initially respond, and nearly all of them eventually develop resistance, which represents an important barrier to long-term survival.

In this issue of *Cancer Drug Resistance*, Rajendran *et al.*^[14] ^elegantly reviewed the mechanisms of cisplatin activity and resistance in BC and the potential of bioactive natural compounds, such as phytochemicals and flavonoids, to overcome this resistance and improve therapeutic response. As they pointed out^[14]^, resistance to cisplatin is a complex, multifactorial process that can be attributed to specific mechanisms intrinsic to BC biology or to general mechanisms common to different tumor types or drug pharmacokinetics. Several factors have been associated with resistance to cisplatin, including decreased intracellular drug concentration mediated by drug efflux pumps, alterations in DNA repair genes, and increased drug cytosolic inactivation^[15]^. I fully support their argument^[14]^ that a greater understanding of the mechanisms of cisplatin activity and resistance, as well as the identification of potential biomarkers of response, is required so that we can select patients likely to respond to cisplatin and to improve strategies to fight resistance mechanisms. One such strategy is based on targeting the effectors and pathways involved in chemoresistance with natural compounds that can potentially synergize with platinum drugs, such as cisplatin. The antitumor effects of natural products are generally attributed to suppression of cell proliferation and metastasis, induction of apoptosis, and stimulation of autophagy, with minimal side effects^[16]^.

Based on our group’s experience in different tumor types, including mBC^[17-19]^, we can highlight the potential and promising role of curcumin and its derivatives as chemosensitizing agents^[20]^. Curcumin (*diferuloylmethane*) is a hydrophobic polyphenol. It is the major curcuminoid in the spice turmeric (77% curcumin, 17% demethoxycurcumin, and 3% bis-demethoxycurcumin) and is derived from the rhizome of the herb *Curcuma longa*. Many of curcumin’s antitumor properties have been attributed to its role as an anti-inflammatory and antioxidant or to its ability to modulate the cell cycle, several pathways, and transcription factors involved in proliferation, apoptosis, migration, invasion, angiogenesis, metastasis, and chemoresistance. Moreover, it has a very low toxicity profile in humans^[20]^. In particular, several studies have demonstrated that curcumin can enhance cisplatin sensitivity and prevent drug resistance in different tumor types^[21]^. Interestingly, Park *et al.*^[21]^ demonstrated that co-treatment with curcumin and cisplatin synergistically induced apoptosis through ROS-mediated activation of the MEK/ERK pathway and the downregulation of survival proteins in BC cell lines and xenograft mouse models. More recently, it has been shown that the curcuminoid demethoxycurcumin enhanced the chemotherapeutic efficacy of cisplatin on HER2-overexpressing BC cells and inhibited the HER2-induced drug resistance of tumor cells through the inhibition of the PI3K/AKT signaling pathway^[22]^. Furthermore, it is well known that a major obstacle for the clinical use of cisplatin is its associated toxicities, including renal damage, deafness, and peripheral neuropathy, which negatively affect the overall efficacy of treatment^[15]^. Interestingly, curcumin not only increases the efficacy of cisplatin in several cancer models but also decreases cisplatin-related toxicity^[16,23,24]^. The potential clinical scenarios for the combined treatment of cisplatin plus curcumin in BC are depicted in Figure 1.

**Figure 1:**
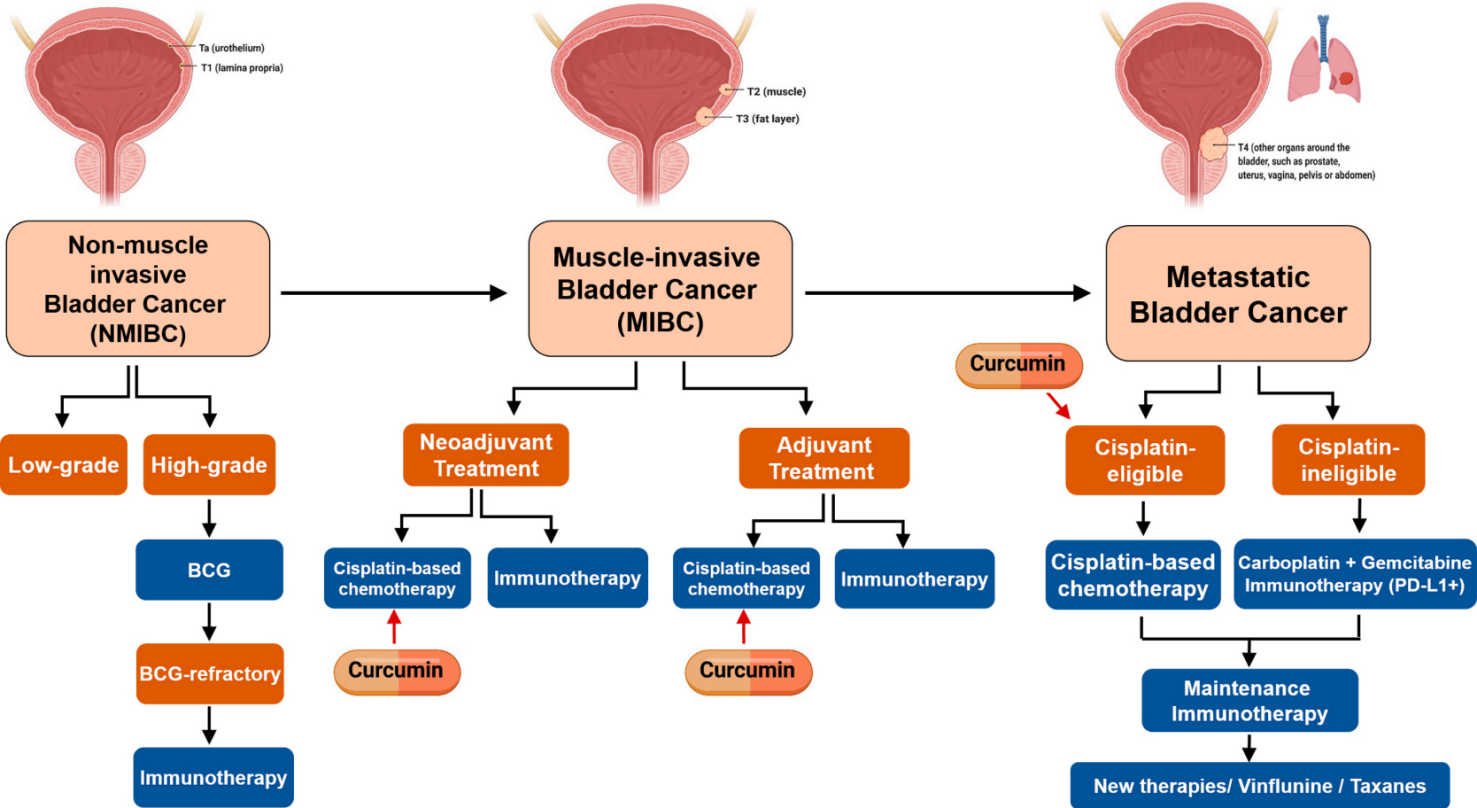
Potential clinical settings for the combined treatment of cisplatin plus curcumin in BC. Created with BioRender.com. BC: Bladder cancer.

However, although it is true that *in vivo* and *in vitro* data are promising, the clinical application of these naturally derived bioactive compounds has been limited by their poor bioavailability in humans due to poor absorption and water solubility as well as to their rapid metabolism and systemic elimination^[25,26]^. Nevertheless, to solve this problem, in recent years, many efforts have been focused on obtaining new promising bioavailable formulations or delivery strategies: liposomes, micelles, phospholipid complexes, microemulsions, nano-emulsions, emulsions, solid lipid nanoparticles, nanostructured lipid carriers, biopolymer nanoparticles, and microgels^[27,28]^. Nonetheless, as proposed by Rajendran *et al.*^[14]^, high quality pharmacokinetic and pharmacodynamic studies are required for proper dosing of these new compounds. Moreover, increasing our understanding of how these compounds work is critical to developing synthetic derivatives with improved pharmacokinetics and greater bioavailability and efficacy.

In conclusion, although cisplatin is a clinical mainstay for the treatment of MIBC, many tumors unfortunately develop resistance and are refractory to treatment. As Rajendran *et al.*^[14]^ pointed out, combination therapies using natural products represent a promising effective and novel strategy to overcome this resistance. For instance, the combination of curcumin and cisplatin could be a potential synergistic strategy to attenuate cisplatin-related adverse effects and decrease resistance. Finally, improved delivery strategies and new formulations offer encouraging ways to increase the bioavailability of these natural products. Hopefully, these strategies will be tested in further randomized, double-blind, placebo-controlled clinical trials in BC patients and incorporated into clinical practice. Nevertheless, although clinical trials using new bioavailable curcumin formulations are certainly mandatory to evaluate the optimal dosage, safety, and antitumor activity, based on our group’s previous preclinical results^[19] ^as well as on those presented by Rajendran *et al.*^[14]^, I suggest that the combination of curcumin plus a platinum agent, such as cisplatin, could be an effective and reliable approach for the management of several cancers, including BC.

## DECLARATIONS

### Copyright

© The Author(s) 2022.
